# Nuclear lamin A in rotator cuff tear margin tenocytes: an antiapoptotic and cell mechanostat factor

**DOI:** 10.1186/s13018-021-02569-1

**Published:** 2021-06-30

**Authors:** Stefano Gumina, Barbara Peruzzi, Martina Leopizzi, Natale Porta, Valeria Di Maio, Carlo Della Rocca, Vittorio Candela

**Affiliations:** 1 Department of Anatomical, Histological, Forensic Medicine and Orthopaedics Sciences, Sapienza University of Rome, Istituto Clinico Ortopedico Traumatologico (ICOT), Latina, Italy; 2 Multifactorial Disease and Complex Phenotype Research Area, IRCCS Bambino Gesù Children’s Hospital, Rome, Italy; 3 Department of Medico-Surgical Science and Biothecnologies, Sapienza University of Rome, Polo Pontino, Latina, Italy

## Abstract

**Background:**

The network of intermediate filament proteins underlying the inner nuclear membrane forms the nuclear lamin. A- and B-type lamins are the major components of the nuclear lamina. Lamins function in many nuclear activities. The role of lamin A and transcription factors (NF-kB) as anti-apoptotic is well documented. Recently, lamin A has also been considered as a mechanosensor protein that is able to maintain nuclear integrity from mechanical insults.

We aimed to verify how lamin A expression varies in healthy cuff cells and in those with different-sized tears where various mechanical stresses are present.

**Methods:**

Forty-three patients with rotator cuff tear (RCT) [23M–20F, mean age (SD): 63.5 (6.1)] were enrolled. Tissue samples excised from the most medial point of tear margins were analyzed for lamin A expression by immunohistochemistry. Controls were represented by samples obtained by normal supraspinatus tendons excised from patients submitted to reverse shoulder prosthesis implant [8M–7F, mean age (SD): 67.9 (7.1)]. The intensity of staining was graded, and an H-score was assigned. Statistical analysis was performed.

**Results:**

Our study revealed a moderate intensity of lamin A in the healthy cuff tendons, a higher expression of this protein in the small tears, and a significant decrease of lamin A with increasing tear size (p < 0.0001).

**Conclusions:**

Our study emphasizes the importance of early repair of small RCTs since nuclear stability is maintained, and the cellular function is protected by lamin A overexpression. High re-tear of massive cuff repair could be due to cellular apoptosis and nuclear modifications induced by lamin A lack.

**Level of evidence:**

III

## Introduction

In eukaryotic cells, the nuclear envelope separates chromosome and subnuclear structures from the cytoplasm and regulates the trafficking of macromolecules, including proteins, DNAs, and RNAs, between nuclear and cytoplasmic compartments. This transport takes place across nuclear pore complexes that are spaced across the nuclear envelope. The latter is constituted by a double-membrane structure consisting of an inner and outer nuclear membrane. Underlying the inner nuclear layer is a network of intermediate filament proteins forming the nuclear lamina (20–50 nm thick) [1]. The lamina structurally supports the nuclear envelope, determines the overall shape of the interphase nucleus, and provides an anchoring site for nuclear pore complexes [2]. In vertebrates, A-type and B-type lamins are the significant components of the nuclear lamina. Both are type V intermediate filament proteins that are found exclusively in the nucleus. The two subtypes (A- and B-type lamins) are distinguished by their protein sequences, physical properties, and expression profiles [2]. In humans, A-type lamin is encoded by the LMNA gene, while two separate genes (LMNB1 and 2) encode B-type lamins. By alternative splicing, the LMNA gene also produces lamin C isoforms lacking the final two exons found in lamin A [3].

Lamins function in many nuclear activities such as DNA replication [4], chromatin organization [5]; transcriptional regulation, nuclear shape maintenance, and signal transduction [6]; and nuclear stability [7].

Recently, Swift et al. [8] have also attributed to lamin A a role as mechanosensor protein, focusing their attention on the correlation between lamin protein levels and the extracellular matrix stiffness and mechanics. A recent biomechanical study [9] conducted on healthy and injured cuff tendons belonged to adult rats. It has been observed that the defect group exhibited reduced stiffness, reduced ultimate load, and reduced area under the curve at ultimate stress compared to the intact tendon group. Furthermore, the authors stated that transverse strain increased with increasing axial load in the defect group region but did not change for the intact group. How this is translated into the cuff tendon cell is not yet known. Since the role of A-type lamins in maintaining nuclear structural integrity from mechanical insults and cell viability has been demonstrated [10, 11], we have conducted an immunohistochemical study to verify how A-type lamin expression varies in healthy cuff tendon cells and in those with different sized tears where inevitable different mechanical stresses are present.

## Material and methods

Fifty-four consecutive patients [25M–29F, mean age (SD): 65.2 (5.18)] with rotator cuff tear (RCT) were enrolled. The diagnosis was obtained after physical examination, standard X-ray (true AP and axillary views), and MRI of the involved shoulder. Exclusion criteria were as follows: traumatic RCT; anti-inflammatory drug assumption during the 2 months before surgery; and V-shaped or L-shaped lesions, diabetes, rheumatologic diseases, and prior surgery.

Tissue samples excised from the most medial point of tear margins [12] were analyzed for lamin A expression by immunohistochemistry. Fifteen normal supraspinatus tendon biopsies, excised in patients [8M–7F, mean age (SD): 67.9 (7.1)] with proximal humerus fractures with intact rotator cuff submitted to reverse shoulder arthroplasty, were used as controls. In fact, supraspinatus tendon is usually sacrificed in patients submitted to reverse shoulder arthroplasty. Furthermore, all controls were submitted to a preoperative CT scan which confirm the health status of the rotator cuff muscle status.

The Southern California Orthopaedic Institute (SCOI) [13] classification of complete RCTs was used to classify tendon tears intraoperatively. We considered the lesions belonging to type I as small, those of types II and III as large, and those of type IV as massive.

Immunohistochemistry was conducted on RCT sections by following the manufacturer’s instructions with HRP-DAB-based kit (Dako LSAB Kit peroxidase; Dako, Carpinteria, CA). RCT sections were deparaffinized and rehydrated in graded ethanol. Endogenous peroxidase activity was blocked by 3% hydrogen peroxide. Antigen retrieval was performed in EDTA buffer pH 8.0 (UCS Diagnostic). The sections were then incubated overnight at 4 °C with a rabbit anti-lamin A/C antibody (A0249, Abclonal; Woburn, MA) and subsequently stained using Dako LSAB Kit peroxidase.

For each sample, the nuclear expression of A-type lamin in tenocytes was evaluated by a pathologist using two criteria: the percentage of immunoreactive cells (semiquantitative method) and the intensity of staining (qualitative method). The percentage of immunoreactive cells was estimated by a medium-magnification (20×) microscope examination of the entire section. The intensity of staining was graded as follows: negative (0), low staining (1), moderate staining (2), and high staining (3). The percentage of cells at each staining intensity level was calculated, and an H-score was assigned using the following formula: [1 × (% cells 1+) + 2 × (% cells 2+) + 3 × (% cells 3+)]. The final score, ranging from 0 to 300 (H1 = 0–50; H2 = 51–100; H3 = 101–150; H4 > 150), gives more relative weight to higher intensity staining in a sample.

All participants signed an informed consent form in accordance with the Declaration of Helsinki; the study obtained the local ethics committee approval (Prot xt/2020).

### Statistical analysis

All analyses were completed using GraphPad Prism 6.0. Contingency table data were analyzed by Fisher’s exact (chi-square) test of independence. Accordingly, to chi-square analysis requirements, the score 0 of A-type lamin intensity (negative staining) was excluded by chi-square analysis since no samples resulted negative. The level of significance was set at alpha = 0.05. SPSS 20 (IBM, Chicago, IL) for Windows was used.

## Results

The studied group was finally composed of 43 cases [23M–20F, mean age (SD): 63.5 (6.1)] and seven controls [8M–7F, mean age (SD): 67.9 (7.1)]. RCTs were intraoperatively classified in 21 small, 14 large, and 8 massive tears.

**Fig. 1:**
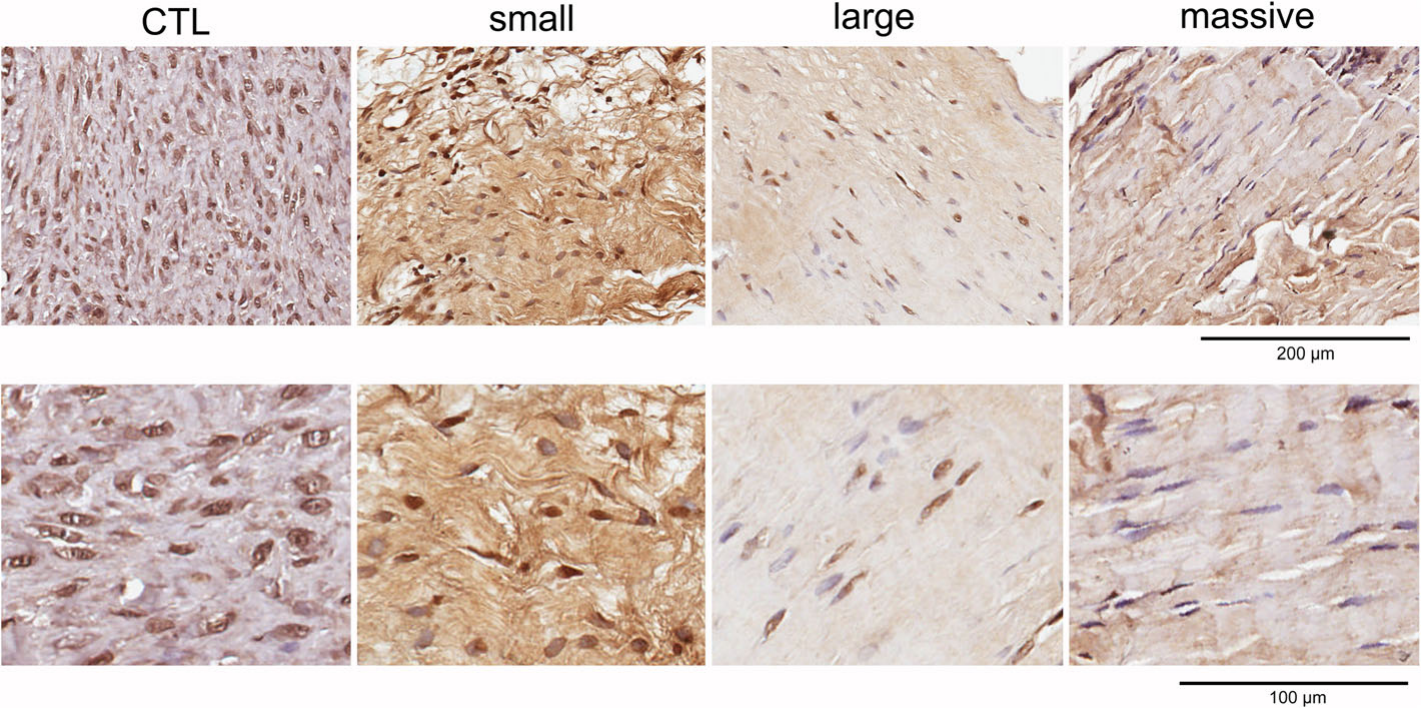
Immunohistochemical staining of control (CTL) and rotator cuff tear samples for lamin A/C. Nuclei are stained by hematoxilin dye. Scale bar: 200 μm (upper panels) and 100 μm (lower panels)

A panel of lamin A immunohistochemical staining is shown in Fig. 1.

**Fig. 2:**
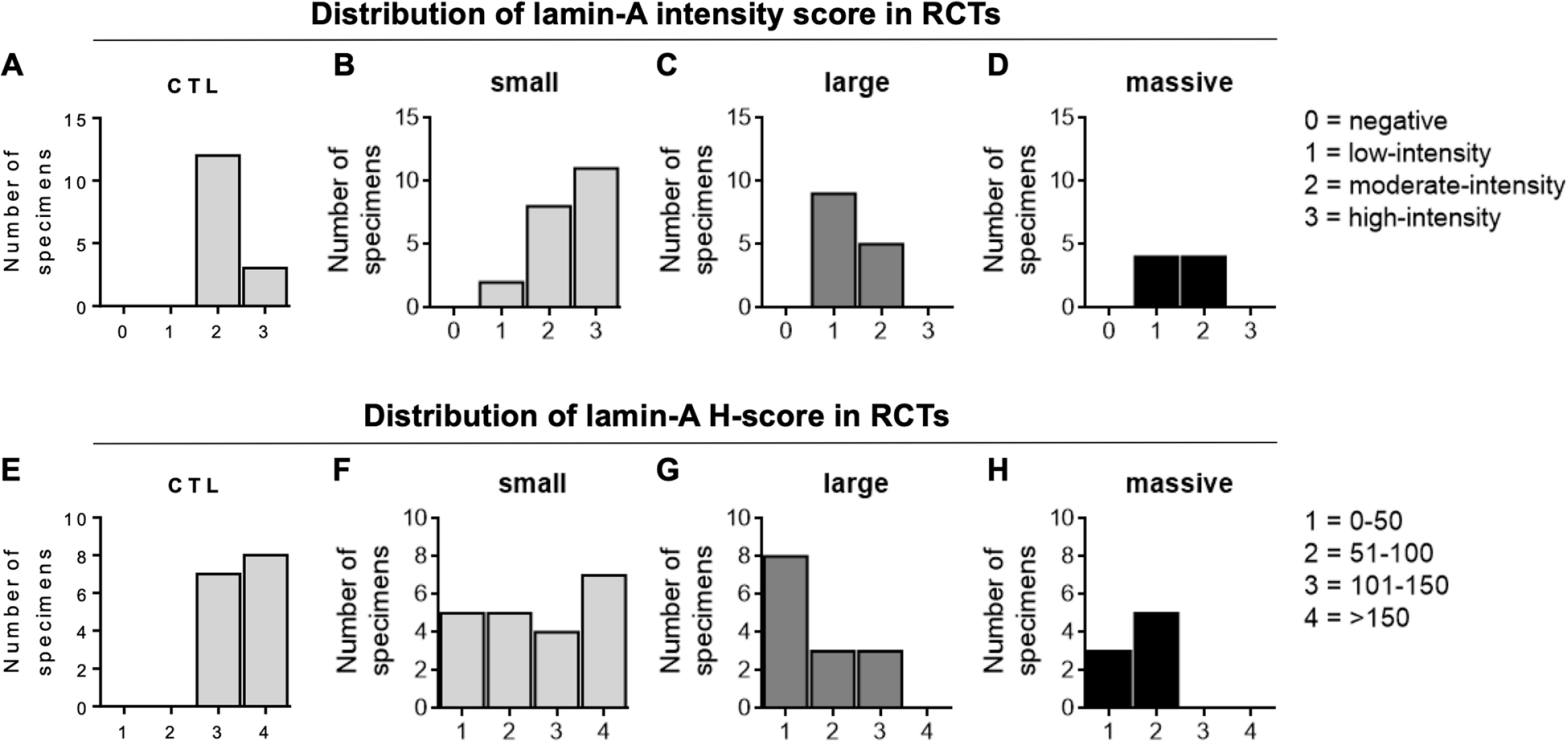
Distribution of lamin A/C immunohistochemical staining intensity (upper panels) and H-scores (lower panels) in controls (**A**, **E**), small (**B**, **F**), large (**C**, **G**), and massive (**D**, H) rotator cuff tear specimens

The intensity of staining and the H-score of immunoreactive cells, assessed by microscope analysis, are shown in Fig. 2. As regarding the nuclear intensity score of lamin A immunoreactive cells, no negative samples were counted. Analysis of the distribution of the intensity staining scores showed the 20% of high-stained and the 20% of moderate-stained samples for control tendon samples (Fig. 2A), 52% of high-stained, 38% of moderate-stained and 10% of low-stained samples for the small RCTs (Fig. 2B), 36% of moderate-stained and 64% of low-stained samples for the large RCTs (Fig. 2C), and 50% of both moderate- and low-stained samples for the massive RCTs (Fig. 2D). Analysis of the H-score distribution showed 53% of H4-score and 47% of H3-score for control tendon samples (Fig. 2E); 33% of H4-score, 19% of H3-score, 24% of H2-score samples, and 24% of H1-score for the small RCTs (Fig. 2F); no H4-score samples, 21% of H3-score, 21% of H2-score samples, and 57% of H1-score for the large RCTs (Fig. 2G); and no H4- and H3-score samples, 63% of H2-score samples, and 38% of H1-score for the massive RCTs (Fig. 2H).

The chi-square test showed a statistically significant correlation between the nuclear expression of A-type lamin and the size of the RCTs (Table 1).

**Table 1:** Results of chi-square analysis of lamin A intensity staining and H-score in RCT patients and control (CTL) tendons

Samples	Lamin A intensity staining				Lamin A H-score				
	1	2	3	***p*** value	1	2	3	4	***p*** value
CTL	0	12	3	**< 0.0001**	0	0	7	8	**< 0.0001**
Small	2	8	11		5	5	4	7	
Large	9	5	0		8	3	3	0	
Massive	4	4	0		3	5	0	0	

Lamin A intensity staining: 1: low-intensity; 2: moderate-intensity; 3: high-intensity

Lamin A H-score: 1: 0–50; 2: 51–100; 3: 101–150; 4: 151–200

## Discussion

The skeletal anatomy’s potential importance on the genesis of rotator cuff tear has been studied for decades and remains a controversial subject. Recently, tendon degeneration (age-related or degeneration induced by genetics and medical conditions) seems to be the most credited theory for cuff rupture [14–17]. The origin of RCT is represented by an area of the tendon within a few mm of its insertion characterized by relative hypovascularization [18]. The inadequate blood supply is only partially improved by vascular anastomoses of the critical zone, near the tendon insertion. Micro vascularization may become worse in many patients with smoking and alcoholic habit [19, 20], hypertension [21], thyroid pathology [22], and lung and other cardiovascular diseases [23]. Obesity is also considered a risk factor for rotator cuff tear [24] because it contributes to peripheral vascular deficiencies through its associations with an increased production of adipokines (leptin; adiponectin; plasminogen activator inhibitor; tumor necrosis factor-a; angiotensinogen; interleukins 6, 8, 10, and 18). All these molecules induce oxidative stress, inflammation, thrombosis, and endothelial dysfunction [25]. The consequent release of many reactive oxygen species (ROS) may lead to degeneration of the tendon causing oxidative stress and cell apoptosis [25, 26].

When cuff tear occurs, multiple stimuli, both mechanical and inflammatory, lead to the altered expression of proteins; some of them are probably synthesized as an attempt to tendon healing. In this regard, increasing in periostin on rotator cuff margins was attributed to the attempt of the tendon to change its viscoelastic properties to prevent increased damage [20]. Our study revealed a moderate intensity of type V intermediate filament proteins lamin A in the healthy cuff tendons (control group), a higher expression of this protein in the small tears, and a significant decrease of lamin A presence with increasing tear size. Since it was demonstrated that cells lacking lamin A have elevated ROS levels [26, 27], it is plausible that the initial increase in lamin A observed in our specimens that belonged to patients with small rotator cuff tear is ascribable to an attempt to face up ROS levels. However, this attempt is destined to wane given the progressive cell depletion observed in large and massive tears.

In an immunohistochemical analysis on rotator cuff tear margins, nuclear transcription factors as NF-kB have been observed [28]. Furthermore, activated NF-kB on the tear edge increases with increasing tear size. The role of lamin A and of NF-kB as anti-apoptotic is well documented [2, 29–31], such as it is well known that the same factors that regulate NF-kB activation act as neoangiogenesis inductors [28]. Interactions between lamin and nuclear transcription factors have been demonstrated in vivo and in vitro [31, 32]. We hypothesize that the increased expression of lamin A in the nuclei of small tear tendon tenocytes is initially related to the NF-kB activation. Therefore, lamin A’s action as anti-apoptotic would occur not only through the protective effect that lamin carries out on the cell nucleus [7, 8, 10] but also through the regulation of the NF-kB.

A proteomic analysis conducted by Swift and Discher [33] revealed that, in vitro, nuclei of mesenchymal cells on a soft substrate are wrinkled and relaxed. In contrast, on the stiff substrate, they are flattened by stress fibers and appear taut and smooth. Furthermore, the native fold of lamin A is maintained on a stiff substrate, but the total quantity of lamin A protein is upregulated. Lammerding et al. [27] observed that the viability of fibroblasts lacking lamin A was significantly reduced under mechanical strain, while under unstrained conditions were similar to wild-type fibroblasts. Our study revealed a moderate intensity and a high H-score of lamin A in the normal cuff, Instead, the chi-square analysis documented a statistically significant correlation between the nuclear expression of lamin A and rotator cuff tear size; in particular, the greatest expression of lamin A was found in the small tears with a significant decrease in lamin A presence with increasing tear size. Increased lamin A levels, in response to extracellular tension, prevent distortion of the nucleus by physical stress, thereby identifying lamin A as a mechanostat factor in cells [29]. Andarawis-Puri et al. [12], in a biomechanical study, demonstrated that the maximum principal strains were directly medial to the tear, corresponding to the direction of tear propagation and to the site where we performed the biopsies. Locke et al. [9] observed that in rat rotator cuff tendons strain concentrations develop near attachment defects. In a biomechanical study conducted on cadaveric cuff tendons, Miller et al. [34] registered that the largest strain is particularly concentrated medial and posterior to the tear. With increasing tear size, we observed a decrease in lamin A staining and the H score. In massive tears, no 3–4 points H score were found. This may be due to the reduction of the mechanical stress acting on an extended margin of the tear (and not in a concentrated area, as in small tears occur) and the progressive fatty infiltration to which the muscle of the involved tendon undergoes. This analysis seems to be supported by the Swift and Discher theory [33], according to which mechanical loading can cause inhomogeneous straining, making it beneficial to have mechanisms of lamin regulation that act at the local level of individual cells.

The “cellular protection” mechanism progressively fails, and programmed cell death develops. However, in massive tears, some functional cells remain, probably due to the permanent high levels of NFkB.

The relatively low number of studied patients represents a limit of our study. However, the evaluation of lamin A immunohistochemical staining is a novel and expensive methodology.

## Conclusions

Our study emphasizes the importance of early repair of small RCTs since nuclear stability is maintained, and the cellular function is protected by lamin A overexpression.

This study provides a possible explanation for the high re-tear/no healing rate of massive rotator cuff repair; in fact, intracellular modifications leading to apoptosis have already been developed.

## Data Availability

The datasets used and analyzed during the current study are available from the corresponding author on reasonable request.

## Abbreviations

RCT: Rotator cuff tear
SCOI: Southern California Orthopaedic Institute
ROS: Reactive oxygen species
